# The correlation between family food handling behaviors and foodborne acute gastroenteritis: a community-oriented, population-based survey in Anhui, China

**DOI:** 10.1186/s12889-018-6223-x

**Published:** 2018-11-26

**Authors:** Yujuan Chen, Yufeng Wen, Jiangen Song, Baifeng Chen, Shushu Ding, Lei Ding, Jiajia Dai

**Affiliations:** School of public health, Wannan Medical College, 22 West Wenchang Road, Wuhu, 241002 Anhui Province China

**Keywords:** Foodborne acute gastroenteritis, Incidence, Correlation, Food handling, Family environment

## Abstract

**Background:**

Foodborne acute gastroenteritis is a significant public health concern. Food handling plays a key role in the risk of foodborne acute gastroenteritis. However, research focused on the correlation between foodborne acute gastroenteritis and food handling in the family environment is limited. The purpose of the current study was to determinate the association between food handling behaviors in the family environment and foodborne acute gastroenteritis.

**Methods:**

A cross-sectional investigation was conducted from September 1, 2015 to August 30, 2016 in Anhui Province, China. A multistage stratified cluster sampling method was designed to select subjects. Data on foodborne acute gastroenteritis and food handling were collected via questionnaire survey.

**Results:**

Of the 1516 subjects included in the study, 165 (10.9%) reported having experienced symptoms of foodborne acute gastroenteritis in the past 4 weeks. The following behaviors were more prevalent in those that experienced acute gastroenteritis: (1) infrequently thoroughly heating milk (75.6%); (2) infrequently thoroughly heating cooked food purchased from outside (71.3%); (3) infrequently thoroughly heating leftovers stored in the refrigerator (32.5%), and (4) infrequently storing leftovers in the refrigerator (41.6%). A multivariate logistic regression analysis found that foodborne acute gastroenteritis was associated with the following behaviors: (1) often eating raw seafood (*P* < 0.001, OR = 3.250, 95% CI = 2.136–4.946); (2) often storing raw meat and cooked meat in the same container (*P* < 0.001, OR = 4.291, 95% CI = 2.722–6.765); (3) infrequently thoroughly heating milk (*P* < 0.001, OR = 4.665, 95% CI = 2.526–8.617); (4) infrequently thoroughly heating leftovers stored in the refrigerator (*P* < 0.001, OR = 3.416, 95% CI = 2.139–5.454); (5) infrequently storing leftovers in the refrigerator (*P* < 0.05, OR = 1.775, 95% CI = 1.169–2.696); and (6) infrequently thoroughly cooking green beans (*P* < 0.001, OR = 2.859, 95% CI = 1.798–4.545).

**Conclusions:**

Poor food handling behaviors in the family environment are associated with foodborne acute gastroenteritis. Infrequent thorough heating and improper food storage are the most critical risk factors in foodborne acute gastroenteritis.

## Background

Foodborne acute gastroenteritis is an important cause of morbidity and mortality, especially in developing countries [1]. Over the past 20 years, the burden of foodborne acute gastroenteritis caused by specific pathogens has been estimated using the combined data obtained from the Foodborne Diseases Active Surveillance Network, community population surveys, or hospital records. However, the global burden of foodborne acute gastroenteritis remains unknown. The World Health Organization reported that there were approximately 600 million foodborne cases with 420,000 associated deaths in 2010 [2]. In the United States, the Center for Disease Control received reports of 13,405 foodborne disease outbreaks between 1998 and 2008, including 273,120 reported cases of foodborne disease, 9109 hospitalizations, and 200 deaths [3]. In Australia, 444 outbreaks of gastroenteritis and foodborne disease were reported in 2003, 99 of these outbreaks were caused by food, resulting in 1686 individual cases, 105 hospitalizations, and nine deaths [4]. In Iran, a total of 2250 gastroenteritis outbreaks were reported from 2006 to 2011 [5]. Several population-based investigations from France, Poland, Italy and Barbados have reported that 2.5, 6.7, 7.9, and 4.9%, of respondents, respectively, experienced acute gastroenteritis [6–9]. In China [10], the proportion of acute gastroenteritis cases reported by respondents was 3.2% from 2010 to 2011.

Microorganisms, chemicals, and parasites are the leading contaminants associated with foodborne acute gastroenteritis, with microorganisms as the most common causative factor [11]. Food contamination can occur at any point along the food production chain including the food preparation process in the family environment. To reduce the risk of foodborne acute gastroenteritis, the entire food production chain must be monitored. Many countries have already adopted government oversite devoted to the reduction of foodborne risk and have decreased the incidence of foodborne acute gastroenteritis. Nevertheless, foodborne disease remains a major public health issue worldwide.

The family environment is the last step in the food handling chain from farms and food processing plants to the table. Research from Australia and New Zealand have reported that approximately 20–50% of foodborne diseases were attributed to the home environment [12]. Approximately 36.4% of foodborne outbreaks reported by the European Union were caused by inappropriate food handling practices in the home [13]. In 2015, the Foodborne Diseases Active Surveillance Network in China received reports of 79 food poisoning incidents in the family, resulting in 1510 individual cases of food poisoning, and four deaths [14]. Understanding food poisoning arising from the home environment, is therefore critical.

In China, microorganisms, chemicals, and poisonous animals and plants are the most commonly reported pathogenic factors leading to foodborne disease [15, 16]. Fruits and vegetables, beans and dairy products, flour and rice foods, meat and meat products, milk products and seafood are the foods most commonly associated with foodborne diseases in China [17]. Residual pesticides and nitrites are the main pathogenic factors found in fruits and vegetables [18]. Food poisoning is also caused by eating undercooked or raw poisonous animals and plants. For example, food poisoning incidents caused by undercooked green beans, and undercooked and raw seafood are frequent in China [19–21]. In addition, leftover foods that have not been properly refrigerated are high risk foods for foodborne diseases [22–24]. The majority of Chinese people drink milk daily due to the high nutritional value. However, outbreaks of food poisoning caused by milk without heating are frequently reported [25–27].

The National Health and Family Planning Commission of the People’s Republic of China reported that inappropriate food handling and storage, cross contamination, and misuse of poisonous foods are the main causes of food poisoning in student cafeterias [28, 29]. The results of a survey on knowledge about, attitudes toward, and the practice of food safety showed that inappropriate food handling, such as cross-contamination, improper defrosting of frozen foods, improper storage time and temperature, and inadequate cooking, are potential risk factors for foodborne diseases [30–32]. However, to date, data on the correlation between foodborne acute gastroenteritis and food handling in the family environment in China are limited. We hypothesized that poor food handling behaviors, including inappropriate heating of foods, such as milk, leftovers, and green beans, inappropriate storage of leftovers, cross contamination of food, eating raw or undercooked food, purchasing stale food, and incomplete disinfection of kitchen utensils are associated with foodborne acute gastroenteritis. The current research aimed to examine correlations between food handling behaviors in the family environment and foodborne acute gastroenteritis.

## Methods

A cross-sectional survey was conducted in Anhui Province, China on the correlation between food handling in the family environment and foodborne acute gastroenteritis among individuals aged 18 and over. The survey was conducted from September 1, 2015 to August 30, 2016. The medical ethics committee of Wannan Medical College, Anhui Province, China approved study procedures. Signed informed consent was obtained from all participants or a legal representative.

### Study populations and sampling design

A multistage stratified cluster sampling method was applied to select subjects in Anhui Province in 2015. Survey areas were selected based on environmental conditions, population size, economic level, culture, and lifestyle. In the first stage, 6 cities (Hefei, Fuyang, Wuhu, Suzhou, Xuancheng, and Chizhou) were selected randomly from a total of 17 cities (Hefei, Wuhu, Ma-anshan, An-qing, Chaohu, Bengbu, Huaibei, Liu-an, Tongling, Huainan, Xuancheng, Chuzhou, Huangshan, Fuyang, Haozhou, Suzhou, and Chizhou) in Anhui Province. One district and one county from each of the six cities was then randomly selected. In the second stage, two streets and two towns were selected randomly from each district and each county, respectively. In the third stage, one community and one administrative village was selected from each street and town, respectively, resulting in a total of 24 sites (12 communities and 12 administrative villages). In the fourth stage, 1600 households were randomly sampled from the 24 sites. Of the 1600 households selected, 84 declined participation, resulting in 1516 (94.8%) households that completed interviews.

### Data collection

Approximately 100 to 150 different households were interviewed each month. Over a 12-month period, 1516 households were surveyed. In each household, the individual celebrating his/her birthday next was selected to participate in the survey. For those younger than 12 years old, the questions were answered by parents or guardians. Interviews were conducted by trained investigators using a questionnaire designed specifically for this study. Data gathered from the questionnaire included: (1)general demographic characteristics; (2) occurrence of acute gastroenteritis over the past 4 weeks; (3) behavior of food purchasing, handling, storage, and consumption. All subjects were asked whether they had experienced diarrhea or vomiting in the four weeks prior to the survey. In cases where diarrhea or vomiting were reported, details with respect to possible causes, the suspected food(s), and the origin of suspected foods were recorded. Information related to the behavior of food purchasing, handling, storage, and consumption were obtained from subjects ≥18 years old, who were the primary food handlers in the family.

### Case definition

Acute gastroenteritis was defined as diarrhea and/or vomiting for a period of at least 24 h within the 4 weeks preceding the interview. Diarrhea was defined as at least three loose-stool bowel movements within a 24-h period. Vomiting was recorded only if it was coupled with at least one other symptom (abdominal pain, nausea, or fever). Cases of diarrhea or vomiting caused by irritable bowel syndrome, colitis, colon diverticulitis, pregnancy, chemotherapy/radiotherapy, medication, food allergy, Crohn’s disease, and excess alcohol were excluded from the data set. Respondents who experienced symptoms of foodborne acute gastroenteritis were referred to as the “case group”. Respondents who did not experience symptoms of foodborne acute gastroenteritis were referred to as the “control group”.

### Statistical analysis

The database was constructed using EpiData 3.1 software (EpiData, Odense, Denmark), and statistical analyses were performed using SPSS Software V.18.0 (SPSS Inc., Chicago, IL, USA). All qualitative data were expressed as frequency (percentage). The incidence of foodborne acute gastroenteritis was calculated by dividing the number of subjects reporting foodborne acute gastroenteritis in the four weeks prior to the survey by the total number of subjects. The proportion of those exposed to inappropriate food handling behavior was calculated as the number of subjects who engage regularly in inappropriate food handling behavior relative to the total number of subjects. A chi-square test was used to compare the following: (1) the incidence of foodborne acute gastroenteritis associated with different socio-demographic characteristics; (2) the proportion of those exposed to inappropriate food handling separated by gender; and (3) the proportion of those exposed to inappropriate food handling among cases of foodborne acute gastroenteritis and among those who did not report illness. Unconditional logistic regression was used to explore the association between food handling and foodborne acute gastroenteritis and to compute odds ratios and 95% confidence intervals (95% CI). Linear regression was used to diagnose the collinearity of independent variables through tolerance value and the variance inflation factor (VIF). A Tolerance value < 0.1 or a VIF- value > 10 was considered indicative of collinearity. A *P*-value < 0.05 was considered significant. Two-tailed tests were conducted.

## Results

### Demographic characteristics of respondents

Males comprised 56.5% of the subjects. Over 60% of subjects were between 18 years and 50 years old. The proportions of subjects aged ≤17 years and ≥ 50 years were 5.5 and 10.1%, respectively. Most of the subjects (79.0%) had a secondary or tertiary education. Almost 57.7% of respondents lived in rural areas and 42.3% in urban areas. Approximately 49.5% of the respondents reported that their annual household income was within the range of 30,000–89,999 CNY (4387–13,161 USD), while 33.7% reported an annual household income greater than 90,000 CNY (13,161 USD). The remaining 17.0% of the subjects reported an annual household income of less than 30,000 CNY (4387 USD). Approximately 19% of the respondents were peasant migrant workers who commuted to the city from the countryside to work in the construction industry for six months or more, while approximately 13.7% of the respondents were peasant farm workers in rural areas. The percentage of respondents working as service staff, medical staff, and laborers (e.g. factory workers) were 10.1, 15.4, and 11.3%, respectively. Finally, teachers accounted for 8.9% of the subjects, while students and children accounted for 4.7% of the subjects.

### Incidence and distribution of foodborne acute gastroenteritis

A total of 281 (18.5%) respondents reported that they had experienced symptoms of acute gastroenteritis in the past 4 weeks. Of these respondents, 165 (10.9%) cases were identified as foodborne acute gastroenteritis. As shown in Table 1. The monthly incidence of foodborne acute gastroenteritis was significantly higher in females (15.3%) than in males (7.5%, *P* < 0.001). The incidence rate of gastroenteritis was significantly different among age groups (X^2^ = 49.264, *P* < 0.001), education level (X^2^ = 11.116, *P* < 0.05), and total family income (X^2^ = 12.033, *P* < 0.01). There was a significant difference in incidence of foodborne acute gastroenteritis between urban (7.6%) and rural (13.3%) residents (X^2^ = 12.017, *P* < 0.01). In addition, students and children had the highest incidence (13.9%) of foodborne acute gastroenteritis, followed by peasant workers (13.1%) and peasants (13.0%), however, this difference did not reach significance (X^2^ = 8.233, *P* > 0.05).

**Table 1:** The incidence and 95% CI of foodborne acute gastroenteritis among participants

	Foodborne gastro-enteritis (n)	Total Participants (n)	Monthly incidence		X^2^	P
			%	(95% CI)		
Total	165	1516	10.9	(9.4–12.6)		
Gender						
male	64	856	7.5	(5.5–9.2)	23.534	< 0.001
female	101	660	15.3	(12.6–18.0)		
Age (years)						
≤ 17	12	76	15.8	(7.9–23.7)	49.264	< 0.001
18~	54	567	9.5	(7.1–12.0)		
30~	45	315	14.3	(10.5–18.1)		
40~	18	405	4.4	(2.5–6.4)		
≥ 50	36	153	23.5	(17.0–30.7)		
Education						
primary school and below	36	381	11.3	(7.9–14.8)	11.116	0.011
junior high school	49	387	12.7	(9.6–16.0)		
high school	60	476	12.6	(9.5–15.5)		
bachelor and above	20	335	6.0	(3.6–8.7)		
Total annual income (CNY)						
< 30,000	31	259	12.0	(8.1–15.8)	12.033	0.007
30,000–49,999	31	299	10.4	(7.0–14.0)		
50,000–89,999	65	452	14.4	(11.3–17.7)		
≥ 90,000	38	506	7.5	(5.3–9.2)		
Residence						
urban	49	641	7.6	(5.9–9.8)	12.017	0.001
rural	116	875	13.3	(11.0–15.7)		
Occupation						
students and children	10	72	13.9	(6.9–22.2)	8.233	0.312
service personal	18	153	11.8	(7.2–17.6)		
teacher	9	135	6.7	(3.0–11.1)		
medical staff	18	234	7.7	(4.3–11.5)		
peasant worker	38	290	13.1	(9.3–16.6)		
peasant	27	207	13.0	(8.7–17.4)		
worker	18	171	10.5	(5.8–15.2)		
other or unknown	27	254	10.6	(7.5–14.6)		

### Suspected food and food origin

In the current study, foods suspected of causing illness were divided into eight categories, including: (1) meat and meat product; (2) milk and dairy products; (3) egg and egg products; (4) aquatic products; (5) cereals and cereal products (including rice, wheat flour, maize, mille, buckwheat and other starchy foods such as potato, sweet potato, and taro); (6) beans and bean products; (7) vegetable oil; and (8) fruits and vegetables. As shown in Table 2, the most frequently suspected food was meat and meat products (58.2%), followed by milk and dairy products (17.6%) and aquatic products (10.9%). The most common location of suspected food origin was home (40.6%), followed by street food stalls (24.8%), and fast food restaurants (15.2%).

**Table 2:** Constituent ratio of suspected food and food origin

Suspected food and food origin	Cases (n)	Constituent ratio (%)
Suspected food		
meat and meat products	96	58.2
milk and dairy products	29	17.6
egg and egg products	9	5.5
aquatic products	14	10.9
cereals and cereal products	0	0.0
beans and bean products	9	5.5
vegetable oil	0	0.0
fruits and vegetables	6	3.6
other and unknown	2	1.2
Origin		
home	67	40.6
hotels	5	3.0
fast food restaurants	25	15.2
food supermarket	18	10.9
street food stalls	41	24.8
other and unknown	9	5.5

**Table 3:** Comparison of food handling behaviors between male and female respondents

Behavior	Total n (%)	Male n (%)	Female n (%)	X^2^	P
Often thoroughly heat cooked food purchased from outside before eating					
no	1026 (71.3)	621 (76.7)	405 (64.3)	26.518	< 0.001
yes	414 (28.7)	189 (23.3)	225 (35.7)		
Often thoroughly heat milk before drinking					
no	1089 (75.6)	621 (76.7)	468 (74.3)	1.090	0.297
yes	351 (24.4)	189 (23.3)	162 (25.7)		
Often store leftovers in the refrigerator					
no	468 (32.5)	225 (27.8)	243 (38.6)	18.820	< 0.001
yes	972 (67.5)	585 (72.2)	387 (61.4)		
Often thoroughly heat leftovers stored in the refrigerator before eating					
no	599 (41.6)	459 (56.7)	140 (22.2)	173.063	< 0.001
yes	841 (58.4)	351 (43.3)	490 (77.8)		
Often thoroughly clean raw vegetables or meat purchased from outside					
no	285 (19.8)	177 (21.9)	108 (17.1)	4.950	0.026
yes	1155 (80.2)	633 (78.1)	522 (82.9)		
Often eat raw seafood					
no	1224 (85.0)	684 (84.4)	540 (85.7)	0.448	0.507
yes	216 (15.0)	126 (15.6)	90 (14.3)		
Often store raw meat and cooked meat in the same container					
no	1171 (76.4)	630 (77.8)	541 (85.9)	15.288	< 0.001
yes	269 (23.6)	180 (22.2)	89 (14.1)		
Often thoroughly heat green beans prior to eating					
no	249 (17.3)	184 (22.7)	65 (10.3)	38.091	< 0.001
yes	1191 (82.7)	626 (77.3)	565 (89.7)		
Often thoroughly boil kitchen utensils to disinfect					
no	1039 (72.2)	594 (73.3)	445 (70.6)	1.284	0.257
yes	401 (27.8)	216 (26.7)	185 (29.4)		
Often buy expired packaged food in supermarkets or shops					
no	1053 (73.1)	585 (72.2)	468 (74.3)	0.768	0.381
yes	387 (26.9)	225 (27.8)	162 (25.7)		
Often Purchase stale raw vegetables, meat and other ingredients					
no	1023 (71.0)	540 (66.7)	483 (76.7)	17.222	< 0.001
yes	417 (29.0)	270 (33.3)	147 (23.3)		
Often eat outside the home					
no	1238 (86.0)	698 (86.2)	540 (85.7)	0.062	0.804
yes	202 (14.0)	112 (13.8)	90 (14.3)

**Table 4:** Comparison of food handling behaviors between the case group and the control group

Behavior	Group		X^2^	P
	Case [n (%)]	Control [n (%)]		
Often thoroughly heat cooked food purchased from outside before eating				
no	117 (76.5)	909 (70.6)	2.278	0.131
yes	36 (23.5)	378 (29.4)		
Often thoroughly heat milk before drinking				
no	135 (88.2)	954 (74.1)	14.768	< 0.001
yes	18 (11.8)	333 (25.9)		
Often store leftovers in the refrigerator				
no	63 (41.2)	405 (31.5)	5.875	0.015
yes	90 (58.8)	882 (68.5)		
Often thoroughly heat leftovers stored in the refrigerator before eating				
no	95 (62.1)	504 (39.2)	29.597	< 0.001
yes	58 (37.9)	783 (60.8)		
Often thoroughly clean raw vegetables or meat purchased from outside				
no	33 (21.6)	252 (19.6)	0.341	0.560
yes	120 (78.4)	1035 (80.4)		
Often eat raw seafood				
no	99 (64.7)	1125 (87.4)	55.298	< 0.001
yes	54 (35.5)	162 (12.6)		
Often store raw meat and cooked meat in the same container				
no	91 (59.5)	1080 (83.9)	53.764	< 0.001
yes	62 (40.5)	207 (16.1)		
Often thoroughly heat green beans prior to eating				
no	47 (30.7)	202 (15.7)	21.581	< 0.001
yes	106 (69.3)	1085 (84.3)		
Often thoroughly boil kitchen utensils to disinfect				
no	113(73.9)	926(72.0)	0.247	0.619
yes	40(26.1)	361(28.0)		
Often buy expired packaged food in supermarkets or shops				
no	108(70.6)	945(73.4)	0.561	0.454
yes	45(29.4)	342(26.6)		
Often Purchase stale raw vegetables, meat and other ingredients				
no	105(68.6)	918(71.3)	0.485	0.486
yes	48(31.4)	369(28.7)		
Often eat outside the home				
no	123(80.4)	1115(86.6)	4.420	0.036
yes	30(19.6)	172(13.4)

**Table 5:** Logistic regression analysis of risk behavior on foodborne acute gastroenteritis

Factors	B	P	Exp (B) (95%CI)	Collinearity Statistics	
				Tolerance	VIF
Residence				0.774	1.292
urban	Ref.	Ref.	Ref.		
rural	1.041	< 0.001	2.832 (1.880–4.454)		
Gender				0.847	1.181
male	Ref.	Ref.	Ref.		
female	2.082	< 0.001	8.019 (4.885–13.164)		
Often thoroughly heat milk before eating				0.752	1.329
no	1.54	< 0.001	4.665 (2.526–8.617)		
yes	Ref.	Ref.	Ref.		
Often thoroughly heat leftovers stored in the refrigerator				0.752	1.33
no	1.228	< 0.001	3.416 (2.139–5.454)		
yes	Ref.	Ref.	Ref.		
Often store leftovers in the refrigerator				0.783	1.276
no	0.574	0.007	1.775 (1169–2696)		
yes	Ref.	Ref.	Ref.		
Often store raw and cooked meat in the same container				0.880	1.136
no	Ref.	Ref.	Ref.		
yes	1.456	< 0.001	4.291 (2.722–6.765)		
Often thoroughly heat green beans before eating				0.818	1.222
no	1.05	< 0.001	2.859 (1.798–4.545)		
yes	Ref.	Ref.	Ref.		
Often eat raw seafood				0.964	1.101
no	Ref.	Ref.	Ref.		
yes	1.179	< 0.001	3.250 (2.136–4.946)		
Often eat outside the home				0.909	1.101
no	Ref.	Ref.	Ref.		
yes	0.489	0.063	1.631 (0.973–2.734)		
Constant	−7.316	< 0.001	0.001		

*Ref* Reference, *VIF* Variance Inflation Factor

### The status of food handling in the family environment

As can be seen in Table 3, from a total of 1440, the proportion of respondents who answered “no” to the items: “often thoroughly heat milk”, “often thoroughly heat leftovers stored in the refrigerator”, “often thoroughly heat cooked food purchased from outside”, “often thoroughly heat green beans”, and who answered “yes” to the item “often eat raw seafood” were 75.6, 41.6, 71.3, 17.3, and 15.0%, respectively. The proportion of respondents who answered “no” to “often thoroughly boil disinfection kitchen utensils” and “often store leftovers in the refrigerator” were 72.2 and 32.5%, respectively. In addition, approximately 29.0% of respondents often purchase stale raw vegetables, meat, and other ingredients. Stale, raw vegetables were defined as those that had been maintained in the market for a prolonged period of time before being purchased, and the edges of the leaves had begun to turn yellow and rot. Approximately 26.9% of respondents reported that they often buy expired packaged food. Almost 23.6% of respondents often store raw meat and cooked meat in the same container, while 19.8% of respondents answered “no” to “often thoroughly clean raw vegetables or meat purchased from the market before cooking”.

Male and female respondents differed significantly in their answers to the following food handling behaviors: “often thoroughly heat cooked food purchased from outside” (X^2^ = 26.518, *P* < 0.001), “often thoroughly heat leftovers stored in the refrigerator” (X^2^ = 173.063, *P* < 0.001), “often thoroughly cook green beans” (X^2^ = 38.091, *P* < 0.001), “often store leftovers in the refrigerator” (X^2^ = 18.820, *P* < 0.001), “often thoroughly clean raw vegetables or meat purchased from outside” (X^2^ = 4.950, *P* < 0.05), “often store raw meat and cooked meat in the same container” (X^2^ = 15.288, *P* < 0.001), and “often purchase stale raw vegetables, meat and other ingredients” (X^2^ = 17.222, *P* < 0.001).

### Comparison of food handling behaviors between the case group and the control group

Of the 1440 respondents, 153 reported experiencing foodborne acute gastroenteritis in the previous four weeks and are referred to as the “case group”. The remaining 1287 respondents are referred to as the “control group”. As seen in Table 4, the proportions of those that answered no to “often thoroughly heat milk” (X^2^ = 14.768, *P* < 0.001), “often store leftovers in the refrigerator” (X^2^ = 5.875, *P* < 0.05), “often thoroughly heat leftovers stored in the refrigerator” (X^2^ = 29.597, *P* < 0.001), and “often thoroughly cook green beans” (X^2^ = 21.581, P < 0.001) and yes to “often eat raw seafood” (X^2^ = 55.298, *P* < 0.001) and “often store raw meat and cooked meat in the same container” (X^2^ = 53.764, *P* < 0.001), were significantly higher in the case group compared to the control group.

### Multivariable logistic regression analysis

A multivariable logistic regression was conducted to analyze the association between food handling behaviors and foodborne acute gastroenteritis. Gender, age, occupation, residence, education, economic status, eating out, and food handling behaviors were independent variables, while foodborne acute gastroenteritis was the dependent variable. As shown in Table 5, after eliminating the effects of demographic characteristics on foodborne acute gastroenteritis, the behaviors entered into the regression were: “often thoroughly heat milk before eating” (*P* < 0.001, OR = 4.665, 95%CI = 2.526–8.617), “often thoroughly heat leftovers stored in the refrigerator before eating” (*P* < 0.001, OR = 3.416, 95%CI = 2.139–5.454), “often store leftovers in the refrigerator” (*P* < 0.05, OR = 1.775, 95%CI = 1.169–2.696), “often store raw and cooked meat in the same container” (*P* < 0.001, OR = 4.291, 95%CI = 2.722–6.765), “often thoroughly cook green beans before eating” (*P* < 0.001, OR = 2.859, 95%CI = 1.798–4.545), and “often eat raw seafood” (*P* < 0.001, OR = 3.250, 95%CI = 2.136–4.946). There were no obvious collinear issues between independent variables.

## Discussion

### Incidence and distribution of foodborne acute gastroenteritis

To our knowledge, this is the first survey of foodborne acute gastroenteritis in a community population in Anhui Province, China. The monthly incidence of foodborne acute gastroenteritis recorded in the current study was 10.9%, which was higher than that reported in Italy (8.9%) [7], France (2.6%) [4], Japan (3.5%), Trinidad and Tobago (5.1%), and British Columbia, Canada (9.2%). In addition, the monthly incidence of foodborne acute gastroenteritis in the current study was greater than that previously reported in Jiangsu (4.4%) and Gansu (8.5%) [33–37] Provinces, China. Differences in illness prevalence may be related to exposure to risk factors, research design, and/or survey method.

Consistent with previous studies from China, the incidence of foodborne acute gastroenteritis differed in gender, age, education, economic level, and residence. In the current study, the incidence of foodborne acute gastroenteritis in women (15.3%) was double that of men (7.5%), subjects aged ≥50 years had the highest incidence of foodborne acute gastroenteritis (23.5%) and higher education (bachelor’s degree or above) was associated with the lowest incidence (6.0%). The incidence of foodborne acute gastroenteritis was lowest (7.5%) among respondents with a high income (total annual income greater than 90,000 CNY) and the incidence of foodborne acute gastroenteritis in rural residents (13.3%) was significantly higher than those living in urban areas (7.6%).

Meat and meat products, milk and dairy products, aquatic products, eggs and egg products, beans and bean products, and fruits and vegetable were the foods most commonly suspected of causing illness. Meat and meat products were the most commonly suspected foods (58.2%). The home (40.6%), street stalls (24.8%), and fast food restaurants (15.2%) were the most frequently reported locations where suspected foods were consumed. These results indicate that food hygiene inspection and food safety management of street stalls and fast-food restaurants must be increased and that information on proper food handling must be emphasized in the family environment.

### Food handling in the family environment

Thorough heating of food, storing leftovers in the refrigerator, and the disinfection of kitchen utensils are critically important behaviors in the prevention of the growth of microorganisms [38, 39]. However, most people are not educated in the proper handling of food. Inappropriate food handling occurs in food production, food processing, storage, and consumption. In the current study, most respondents reported that they infrequently thoroughly heated milk (75.6%) and cooked food purchased from outside the home (71.2%). In addition, as many as 41.6% of respondents reported storing leftovers in the refrigerator infrequently.

The storage and processing of raw and cooked foods together is often the cause of cross contamination. For example, raw meat and cooked meat are often stored in the same container. In addition, foods containing toxic chemicals such as green beans and seafood are the cause of food poisoning outbreaks in China. The consumption of undercooked or raw foods that contain toxic chemicals, such as green beans and raw seafood, has led to many outbreaks of food poisoning [40, 41]. In the current study, 23.6% of respondents reported that they often store raw meat and cooked meat in the same container. Most respondents reported that they often thoroughly cook green beans (82.7%) and do not usually eat raw seafood (85.0%).

These results suggest that inappropriate food handling is prevalent among consumers in the family environment. Previous research has shown that a lack of food safety knowledge is detrimental to the behaviors of food handling [42–44]. These results indicate the importance of increasing education of the typical consumer on proper food handling procedures to produced long-term changes in healthier behaviors.

### Food handling behaviors and foodborne acute gastroenteritis

The present study found differences in food handling behavior between respondents that reported foodborne acute gastroenteritis (case group) and those that did not (control group). The behaviors of infrequently thoroughly heating milk before eating, infrequently storing leftovers in the refrigerator, infrequently thoroughly heating leftovers stored in the refrigerator, often eating raw seafood, often storing raw meat and cooked meat in the same container, and infrequently thoroughly cooking green beans, were 1.2, 1.3, 1.6, 2.8, 2.5, and 2.0 times greater in the case group compared to the control group.

The risk of foodborne acute gastroenteritis was 4.7 times greater in those that responded “no” to “often thoroughly heat milk before eating”. Milk that is not thoroughly heated prior to drinking, can increase the risk of foodborne acute gastroenteritis. Drinking cold milk can stimulate the gastrointestinal tract and cause a negative gastrointestinal reaction [45]. In addition, reports of food quality monitoring from several cities indicate that microorganisms such as *Staphylococcus aurous* have been detected in milk and dairy products sold in stores [46–48]. As such, milk that is not heated prior to drinking may still contain high levels of microorganisms, leading to illness.

Respondents reporting that they do not often store leftovers in the refrigerator or infrequently thoroughly heat leftovers before eating were at a 1.8 and 3.3 times greater risk for foodborne gastroenteritis. Containers used for storage, storage time, and storage temperature are associated with microbial contamination of leftovers. Reusing containers that were used for cooking to store leftovers can cause cross contamination. Rapid bacterial growth occurs in leftovers that are maintained between 4 °C (40 °F) and 60 °C (140 °F) [49], and therefore leftovers must be stored in the refrigerator within 2 h. In addition, leftovers should be heated to at least 74 °C (165 °F) prior to eating in order to reduce the risk of any prior cross contamination and/or inappropriate storage temperature [49].

Infrequently thoroughly cooking green beans before eating was associated with a 2.9 times higher risk of experiencing foodborne acute gastroenteritis in the current study. Green beans can be classified into runner beans (*Phaseolus coccineus*), yardlong beans (*Vigna unguiculata*), hyacinth beans (*Purple Haricot*) and snap beans (*P. var. chinensis Hort.*). As an important crop worldwide, green beans are low in fat, high in protein, and rich in minerals and multiplex vitamins. However, the consumption of green beans is often the cause of foodborne disease outbreaks in China. Green beans contain two main substances that cause food poisoning: saponin and phytohemagglutinin. Saponin and phytohemagglutinin are relatively resistant to heat and can are only destroyed at temperatures above 100 °C (212 °F) for ten minutes [50]. Short cooking time and low cooking temperatures increase the risk of saponin and phytohemagglutinin poisoning.

Respondents reporting that they often eat raw seafood were 3.3 times more likely to experience foodborne acute gastroenteritis. One possible reason why eating raw seafood increases the risk of foodborne diseases is that seafood has been contaminated by *Vibrio parahaemolyticus* before consumption. In China, the detection rate of *Vibrio parahaemolyticus* in seafood is 25–40% [51]. The bacteria *Vibrio parahaemolyticus* is a major pathogen in seafood that can cause foodborne diseases outbreaks. For example, of the 2795 foodborne disease outbreaks between 2003 and 2008, 14% were caused by *Vibrio parahaemolyticus*. Moreover, approximately 28% of reported outbreaks caused by *Vibrio parahaemolyticus* were due by aquatic products [52]. Another possible cause that eating raw seafood increases the risk of foodborne diseases is that seafood eaten contains toxins, such as ciguatoxin, histamine, and saxitoxins which can also lead to foodborne diseases [53].

Food handling in the family environment is a complex behavior. Mishandling of food can occur at each step of the food chain process, from purchasing to cleaning, cooking, consuming, and storage. Consumers often do not follow proper food handling practices. Based on our study results, the following food handling behaviors are suggested. First, foods that are not immediately eaten (such as raw seafood, green beans, milk, and leftovers), must be thoroughly heated prior to eating. Second, to reduce cross contamination, containers for cooking food and storing surplus food must be kept separately. Third, leftover food must be stored in the refrigerator in a timely manner.

### Limitations

While the present study provides valuable data on the risk factors associated with foodborne acute gastroenteritis, there are limitation that must be considered. Recall bias may have affected the study results. The data collected relied on respondents accurately relaying past experiences, prior to the start of the study which may result in recall errors. In particular, data on suspected foods and origin of food may be affected. In addition, respiratory diseases can be associated with symptoms similar to those in acute gastroenteritis and therefore, some cases of acute gastroenteritis may have been incorrectly diagnosed. Some respondents that were included in the study therefore, may have had respiratory disease. There was no exclusion criteria for the “control group”. And some respondents could not be excluded from the “control group”, which may have affected the study results. The “control group” was not paired with the “case group” following some criteria (e.g. gender). The distribution of some variables (e.g. gender) may be disequilibrium between the “case group” and the “control group”.

## Conclusions

Inappropriate food handling behaviors in the family environment were associated with foodborne acute gastroenteritis. The behaviors of infrequently thoroughly heating food before eating, infrequently storing leftovers in the refrigerator, and often storing raw meat and cooked meat in the same container are high-risk behaviors associated with foodborne acute gastroenteritis. Further research is needed to explore the most effective methods of resolving inappropriate food handling behaviors in the family environment.

## Abbreviations

CI: Confidence intervals
CNY: China Yuan
OR: Odds ratios
VIF: Variance inflation factor
